# Fc Receptor-Like Proteins in Pathophysiology of B-cell Disorder

**DOI:** 10.4172/2155-9899.1000427

**Published:** 2016-06-17

**Authors:** Mollie Capone, John Matthew Bryant, Natalie Sutkowski, Azizul Haque

**Affiliations:** Department of Microbiology and Immunology, and Hollings Cancer Center, Medical University of South Carolina, 173 Ashley Avenue, BSB-201, Charleston, SC 29425, USA

**Keywords:** Lymphocyte, Immunoreceptor tyrosine activation, Immunoglobulin, Transmembrane, *Chronic lymphocytic leukemia*

## Abstract

Members of the family of Fc receptor-like (FcRL) proteins, homologous to FcγRI, have been identified by multiple research groups. Consequently, they have been described using multiple nomenclatures including Fc receptor homologs (FcRH), immunoglobulin superfamily receptor translocation-associated genes (IRTA), immunoglobulin-Fc-gp42-related genes (IFGP), Src homology 2 domain-containing phosphatase anchor proteins (SPAP), and B cell cross-linked by anti-immunoglobulin M-activating sequences (BXMAS). They are now referred to under a unified nomenclature as FCRL. Eight different human FCRL genes have been identified, all of which appear to be related to the genes of the immunoglobulin superfamily (IgSF) of cellular adhesion molecules. These type 1 transmembrane glycoproteins are composed of different combinations of 5 types of immunoglobulin-like domains, with each protein consisting of 3 to 9 domains, and no individual domain type conserved throughout all of the FCRL proteins. Ligands for the majority of the FCRLs remain unknown. In general, FCRL expression is restricted to lymphocytes and is primarily expressed in B-lymphocytes, supporting FCRL’s involvement in a variety of immune disorders. Most FCRLs functionally repress B-cell activation; however, they might have dual roles in lymphocyte functions as these proteins often possess immunoreceptor tyrosine activation (ITAM) and inhibitory (ITIM) motif elements. The biological functions of these newly recognized FCRL proteins are just beginning to emerge, and might provide the insight necessary for understanding pathophysiology of lymphocyte disorders and treating different immune diseases.

## Introduction

Many different biological functions including antibody secretion, antigen (Ag) presentation, and proliferation of other immune cells are regulated by B-lymphocytes. Therefore, any type of damage to B-cell functioning typically will result in autoimmunity or lymphoproliferative disorders, such as rheumatoid arthritis, systemic lupus erythematosus, chronic lymphocytic leukemia, hairy cell leukemia, and many others [1]. Associations between autoimmune diseases and lymphoma have also been identified primarily with-B cell lymphomas [2]. The regulation associated with B-cells most often occurs via expression of adaptive and innate-type receptors. The specific role that these receptors play is then determined by the phosphorylation pathways that take place following receptor engagement. Therefore, it is crucial to understand the role of these receptors in any damage to B-cell function.

**Figure 1:**
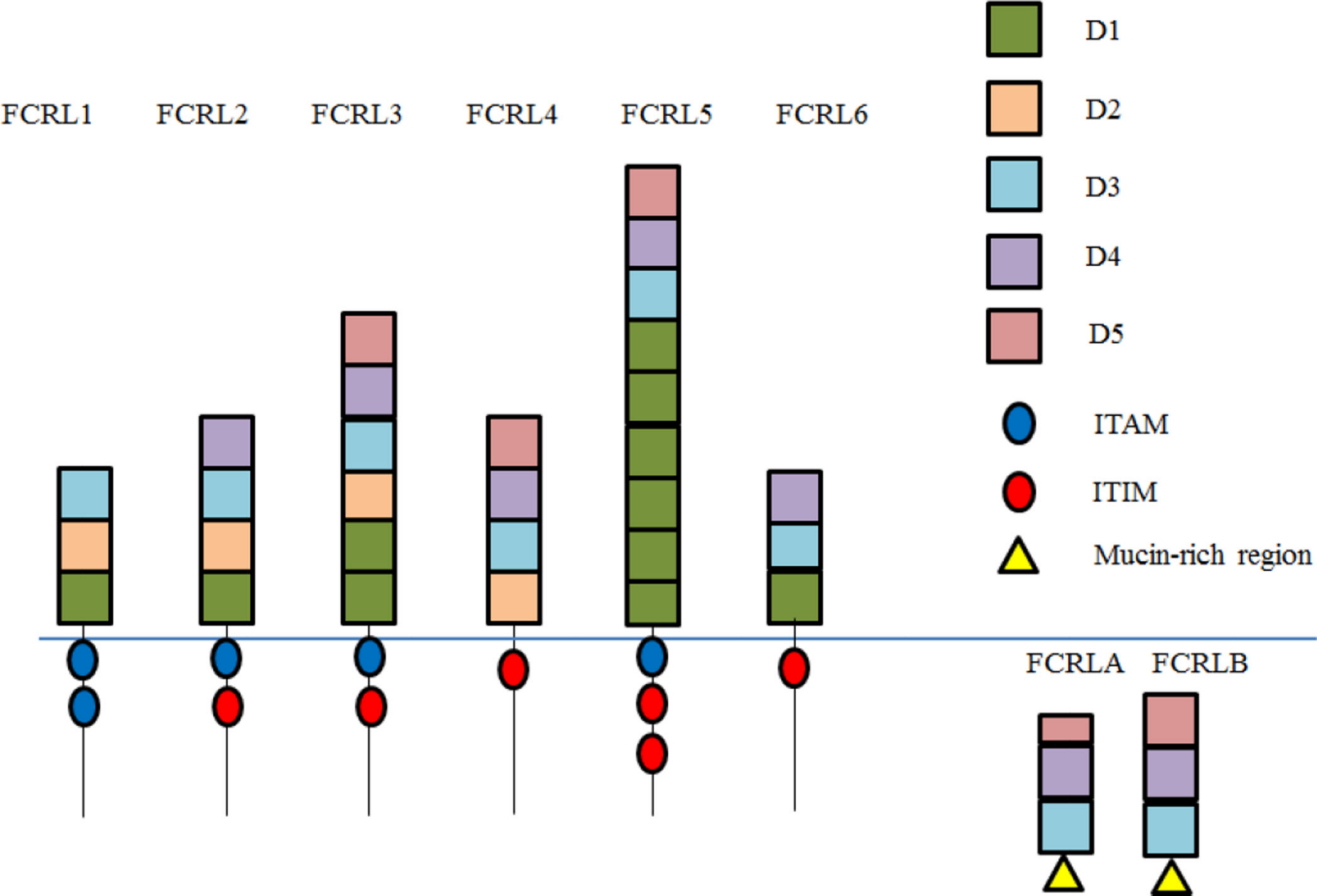
Human Fc receptor-like proteins. The structures for FCRL1–6 as well as FCRLA and FCRLB are represented. Boxes indicate immunoglobulin domains while circles indicate ITAM and ITIM sequences. Mucin-rich regions are also denoted by triangles. The smaller D5 domain on FCRLA refers to the truncated version of the D5 domain found on this protein.

Discovery of new receptors using a number of modern genomic techniques is expanding our understanding of the immune system and the molecules and pathways involved in immunity. Recently, a group of Fc receptor-like (FCRL) proteins have been discovered by multiple groups using different techniques [3–7]. While each group used a different nomenclature, these studies collectively found that the loci for FCRL1 through FCRL5 genes extend over a region of chromosome 1q21-22 approximately 300kB long at a locus which is telomeric of the FCGR1A gene. The FCRL6 gene was then discovered at a locus further telomeric, but proximal to FCER1A. Lastly, the loci for FCRLA and FCRLB genes were located proximal to the FCGR2–3 gene, cumulatively encoding a total of 8 human FcRL proteins [3–7]. The FCRL1–6 genes encode type 1 membrane glycoproteins that contain 3–9 extracellular immunoglobulin (Ig)-like domains. These proteins also contain cytoplasmic regions with ITAM- and ITIM-like elements. In contrast, FCRLA and FCRLB are intracellular proteins that lack any type of transmembrane region, as depicted in Figure 1 [6–8]. The potential for alternative splicing during transcription of the FCRL genes could result in multiple protein isoforms, as was demonstrated by FCRL6 mRNA splicing, where at least 4 different transcripts have been identified that encode 4 distinct FCRL6 isoforms that differ in the number of extracellular Ig-like domains (1–3), and/or in the length of the cytoplasmic tail [9].

The FCRL1 protein consists of three Ig-like domains and contains two ITAM-like sequences in its cytoplasmic tail. FCRL1 expression during B-cell ontogeny is first seen at the pre-B cell stage, and expression intensifies with B-cell maturation [10,11]. It is broadly expressed by mature B-cells, and has also been found on a majority of chronic lymphocytic leukemia (CLL), follicular lymphoma (FL), hairy cell leukemia (HCL), and mantle cell lymphoma (MCL) cells [11–13]; therefore, FCRL1 may be a potential target for immunotherapy of B-cell disorders. FCRL2 is composed of 4 Ig-like domains, and contains one ITAM-like and one ITIM-like sequence in its cytoplasmic tail [6,8]. It is expressed on memory B-cells in the periphery, as well as a circulating marginal zone-like B-cell subset [14,15]. FCRL3 is composed of 6 Ig-like domains with one ITAM-like and ITIM-like sequence. It is found on memory B-cells, marginal zone B-cells, NK cells, CD8^+^ T-cells, and CD4^+^ Treg cells [11,16]. FCRL4 consists of 4 Ig-like domains with a single ITIM sequence. It is expressed in a subpopulation of memory B-cells found in mucosa-associated lymphoid tissues (MALT) [17–19]. FCRL5 is composed of 9 domains with one ITAM-like sequence and two ITIM sequences. It is found on a broad array of B-cells, and is the most highly expressed on terminally differentiated plasma cells [11]. FCRL6 is composed of 3 Ig-like domains, and has a single ITIM sequence. It is expressed by B-cells, by perforin-expressing natural killer (NK) cells, and cytolytic CD8^+^ and CD4^+^ T-cells [20,21]. The intracellular FCRLA and FCRLB proteins are structurally unique, in that they both contain mucin-rich regions, and lack transmembrane and cytoplasmic domains. FCRLA consists of two full Ig-like domains and one truncated domain, and is found in peripheral and tonsil B-cells [22]. FCRLB consists of 3 full Ig-like domains, and seems to be restricted to germinal center B-cells [23,24]. The structural differences found between the various FCRL family members are depicted in Figure 1.

The preferential expression of FCRL proteins on B-lymphocytes, and the presence of ITAM-like and ITIM structural elements in their intracellular domains, suggests that they play a role in the regulation of B-lymphocyte activation. Because most of the FCRL proteins contain both activatory and inhibitory motifs, it is speculated that they are involved in dual modulation. However, initial studies suggested that there was mainly a suppressive function associated with FCRL2–5 proteins, because after BCR cross-linkage, phosphotyrosine (pTyr) activation resulted in the docking of Src homology 2 (SH-2) domain containing phosphatases SHP-1 and/or SHP-2 at the FCRL consensus ITIMs. On the other hand, hints at dual modulation were seen with FCRL3, which appeared to recruit Syk and Zap70 protein tyrosine kinases, in addition to SHP-1 and SHP-2 phosphatases [25]. Furthermore, FCRL3 was later found to promote TRL9-induced B-cell activation, while it suppressed B-cell differentiation into plasma cells.

FCRL1 is the only FCRL family member that contains two ITAM-like sequences and no ITIM sequences; FCRL1 is therefore assumed to be a co-activation receptor. This has been substantiated by the findings that ligation of FCRL1 molecules resulted in B-cell proliferation, while cross-linkage of FCRL1 molecules with the BCR resulted in B-cell activation. So far, ligands have only been identified for FCRL4–6 proteins. FCRL4 was found to bind to heat-aggregated IgA, while FCRL5 was found to bind to heat aggregated IgG. FCRLA was also found to associate with intracellular IgA, IgM and IgG molecules [18]. In contrast, instead of binding to immunoglobulins, FCRL6 was found to bind to MHC class 2/HLA-DR molecules [26]. Discovery of additional FCRL ligands is expected to reveal a great deal more about the involvement of FCRLs in B-cell function.

The role that the FCRL proteins play in B-cell regulation implies a possible role(s) in immune system related diseases and lymphoproliferative disorders. For instance, expression of various FCRLs is up-regulated in Diffuse Large B-cell Lymphoma (DLBCL), FL, and CLL [11–13], while FCRL5 was shown to be upregulated in Burkitt Lymphoma (BL). Furthermore, FCRL6 expression was found to be upregulated in the late stages of HIV-1 infection [9], with FCRL4 similarly upregulated in HIV and hepatitis C infection. In contrast, FCRL3 appears to be involved in autoimmunity [27–29]. Due to their clear associations with disease, FCRLs can be viewed as disease biomarkers, with possible roles as diagnostics or as the targets of immunotherapy. For example, FCRLs 1, 2, 3 and 5 were associated with mutations in immunoglobulin heavy chain variable (IGHV) genes in CLL, with FCRL2 expression strongly predictive of mutation status, >94% concordant with IGHV mutation status [15]. Furthermore, FCRL4 was selectively identified in nodal and extranodal marginal zone lymphomas (MZL), and is considered a positive marker for MZL [30]. Finally, FCRL5 expression is strongly upregulated on the bone marrow plasma cells of multiple myeloma (MM), and it has currently been designated as a target of immunotherapy in MM [31]. A summary of the various FCRL molecules with known associations with human disease is found in Table 1.

While the discovery of the FCRL proteins has shown promise for the prognosis and treatment of diseases [13,32–34], there is still a great amount that is unknown about their roles in the lymphatic system. For example, it is presumed that there are multiple FCRL protein isoforms resulting from alternative splicing of each individual FCRL mRNA. The structure of these FCRL protein isoforms may reveal important information about their biological functions. For instance, 4 protein isoforms of FCRL6 have been identified that differ in the number of extracellular Ig-like domains, and also by the presence of deleted sequences in the cytoplasmic domain [9]. These structural differences would be predicted to influence ligand binding, and/or signaling, respectively. Intraexonic alternative splicing of FCRL2 mRNA is predicted to result in the formation of 2 protein isoforms that differ in the amino acid compositions of domains 3 and 4, which might likewise affect ligand binding [35].

FCRLA was found to have at least 6 alternatively spliced transcripts that would encode markedly different protein isoforms. Originally, it was predicted that the major isoform was a secreted protein [22]; however, it was later determined that FCRLA was retained intracellularly as a resident ER protein that binds IgA, IgM and IgG molecules, possibly acting as a chaperone. More recent data indicates that one of the FCRLA isoforms has a longer signal peptide that allows for efficient secretion of that isoform [36]. Interestingly, the secreted form is not known to bind immunoglobulins. These secreted isoforms of FCRLs might play a vital role in B-cell regulation, although at this time relatively little is known about their function or structure. Considering that FCRL1 and FCRLA are both associated with lymphoma, these secreted forms could potentially serve as targets for immunotherapy. Since a number of the FCRL proteins (and possibly some of their isoforms) still have unknown ligands, it is likely that only minimal amounts of their involvement in B-cell regulation has been uncovered. Overall, the more information that is unearthed about these proteins, the greater their potential to be used in the treatment of various diseases.

## FCRL Structure

### Nomenclature

A unified nomenclature for the FCRL protein family was developed in 2006, because the proteins had been described by different names as a result of being discovered independently by multiple research groups over time [37]. The first FCRL protein to be identified was actually a rat ortholog of FCRL6, a glycosylphosphatidylinositol (GPI)-anchored membrane protein named gp42 that was upregulated on rat NK cells after exposure to IL-2 [38–40]. Gp42 has two Ig-like domains and is thought to be an activation marker because antibodies directed against gp42 on the surface of NK cells stimulated intracellular calcium influx [39]. The first human FCRL genes were identified ten years later in 2001 by the Dalla-Favera group [3]. While identifying the genes found at a chromosomal translocation breakpoint t (1;14)(q21;q32) in multiple myeloma cells, Hatzivassiliou et al. detected the gene corresponding to the FCRL4 protein, which they referred to as IgSF receptor translocation associated gene 1 (IRTA1) [3,4]. They detected a novel fusion protein that had been created by the translocation comprised of sequences encoding the FCRL4 signal peptide fused to the immunoglobulin Cα1 constant region gene encoding the IgA Fc domain. They further identified a total of five FCRL genes on chromosome 1q21-q23, which they named IRTA1-IRTA5 [3,4]. The corresponding FCRL nomenclature associated with these genes is designated in Table 2.

In addition, Davis et al. employed a bioinformatics approach and discovered six members of the FCRL gene family by searching the human genome database with a 32 amino acid consensus motif shared by FcγR1, FcγRII, FcγRIII and the polymeric Ig receptor [5]. They referred to the genes as Fc receptor homologs (FcRH) FcRH1-FcRH6. The corresponding FCRL nomenclature for FcRH proteins is also designated in Table 2. Simultaneously, Xu et al. also discovered two members of the FCRL family of proteins by utilizing *in silico* strategies to search for molecules that had characteristics shared by the IgSF, as well as, Fc receptor and gp42 proteins [41]. They referred to the two new proteins as Src homology (SH)-2 domain-containing phosphatase anchoring proteins SPAP1 and SPAP2, which later became known as FCRL2 and FCRL3, respectively (Table 2). Also at the same time, using subtractive hybridization methods, Nakayama et al. discovered four of the genes for these proteins, which they referred to as B-cell cross-linked by anti-IgM activation sequence (BXMAS) genes [7]. Their corresponding FCRL nomenclature is also listed in Table 2. Guselnikov et al. again identified the family based upon an expressed sequence tag (EST) database search after probing with a consensus sequence corresponding to the unique extracellular domain of FCγR1 [6]. They called the resulting genes IFGPs for their homology to IgSF, FcR, and gp42. The corresponding FCRL names for the IFGP proteins are also listed in Table 2. The same group also identified two additional homologs that had no obvious transmembrane sequences, which they named FCRL1 and FCRL2 [35,42]; these proteins were later renamed FCRLA and FCRLB, respectively [22,37]. These same two proteins were also described simultaneously by the Colonna group as Fc receptor expressed in B-cells FREB [43] and FREB2 [44], and again by the Burrows group as Fc related proteins FcRX [45] and FcRY [46]. In total, 8 different human FCRL family members have been discovered, and in 2006, a unifying nomenclature was proposed, designating the 6 membrane bound human FCRLs as FCRL 1–6, and the two intracellular proteins as FCRLA and FCRLB [22,37].

The unified nomenclature identifies each FCRL based upon its domain structure [37]. FCRL1 corresponds to FcRH1, IRTA5, IFGP1, or BXMAS1. FCRL2 replaces previous names FcRH2, IRTA4, IFGP4, BXMAS2, or SPAP1. FCRL3 was formerly identified as FcRH3, IRTA3, IFGP3, BXMAS3, or SPAP2. FCRL4 was previously referred to as IRTA1, FcRH4, or IFGP2. FCRL5 coincides with IRTA2, FcRH5, IFGP5, or BXMAS. FCRL6 was previously named FcRH6 or IFGP6. FCRLA was adopted for the intracellular protein previously named FCRL, FREB, or FcRX; and FCRLB was adopted for the intracellular protein earlier referred to as FCRL2, FREB2, or FcRY. These changes in nomenclature are summarized in Table 2. Additionally, to unify the naming of FCRL splice variants, they proposed adding the suffix “_v” followed by the number of the variant to the gene name, e.g., FCRL1_v1 for splice variant 1 of FCRL1 gene [37].

### Family member structure

FCRL1–FCRL6 are all type 1 transmembrane glycoproteins that contain immunoglobulin-like domains in their extracellular regions. They also contain cytoplasmic immunoreceptor tyrosine activation motif (ITAM)-like and/or inhibitory motif (ITIM) sequences. Unlike FcRs, which are typically either activatory or inhibitory, three of the FCRLs (FCRL2, FCRL3, and FCRL5) contain both activatory and inhibitory sequences, suggesting that they might be capable of dual-modulation. FCRL1 is the only FCRL family member that contains two ITAM-like regions and is also the only FCRL that contains a charged residue in its transmembrane region where FCRLs 2–6 are hydrophobic and uncharged. FCRL1 also contains three extracellular domains D1, D2, and D3 [6,8]. FCRL2 contains an additional D4 domain and has one ITAM and one ITIM sequence in its cytoplasmic tail. FCRL3 has two D1 domains followed by one of each D2–D5 domains, and like FCRL2, has one ITAM and one ITIM sequence in its cytoplasmic tail. FCRL4, on the other hand, only has one ITIM sequence in its intracellular region and has domains 2, 3, 4, and 5. FCRL5 is the largest of the FCRL proteins with six D1 domains, a D3, D4, and D5 domain. It also has two ITIM sequences and one ITAM sequence in its intracellular region. Finally, FCRL6 has domains D1, D3, and D4 as well as one ITIM sequence in its cytoplasmic tail (Figure 1). FCRLA and FCRLB differ from the 6 main isotypes of FCRL in that they are intracellular proteins and they each contain two to three Ig-like domains. They also lack ITAM or ITIM sequences, and instead have C-terminal mucin-like regions.

### Isoforms

A number of isoforms have been identified for some of the FCRL proteins, and all of the FCRL molecules have the potential to be expressed as a number of different isoforms. FCRL5, FCRL6, and FCRLA have known isoforms. Alternate splicing of FCRL5 generates both secreted and GPI-anchored isoforms along with the typical transmembrane form, and the secreted isoform has been detected at high levels in patients with MM, CLL, and MCL [3]. Through gene expression analysis and cloning of liver fragments, 4 alternative transcripts were discovered for FCRL6, designated FCRL6_v1-v4 variants [9]. The isoform encoded by FCRL6_v1 corresponds to the full length FCRL6 protein, whereas FCRL6_v2 encodes only a single extracellular D5 domain. FCRL6_v3 encodes the D3 and D5 domains, with an altered cytoplasmic tail resulting in an additional tyrosine residue in the C-terminal end. FCRL6_v4 encodes all three extracellular domains, but with a shortened cytoplasmic tail. These studies found that expression of FCRL6_v2 and _v3 transcripts were low in comparison with FCRL6_v1 and _v4 transcripts [9].

The intracellular protein FCRLA has been shown to have seven different isoforms, including a secreted isoform, as suggested by Kulemzin’s group [36]. These isoforms consist of different compositions of the D1–D4 domains that make up the FCRLA protein, and either a short (SSP) or long signal peptide (LSP) that is thought to target it to the ER. The FCRLA locus contains only a single signal peptide exon (SP1), whereas the other FCRL loci were previously shown to contain two exons encoding the signal peptide (SP1 and SP2) [36,47]. However, the EST databases contain some FCRLA cDNAs with 21 base pairs inserted between the D1 domain and the signal peptide sequence, which appears to be an SP2 exon that would produce an FCRLA isoform with a 6 residue longer signal peptide (LSP) [36]. RT-PCR cloning using forward primers that matched either the SP1 or SP2 exons identified the seven alternative FCRLA transcripts [36]. Five of these encoded SSP containing isoforms, while the remaining two encoded LSP containing isoforms. The five SSP isoforms were designated FCRLA4d (containing domains 1, 2, 3, and 4), FCRLA3d (domains 1, 3, and 4), FCRLA2d (domains 3 and 4), FCRLA2d’ (domains 1 and 4), and FCRLA1d (domain 4). The LSP containing isoforms were designated LSP-FCRLA4d and LSPFCRLA2d, and had the same domains as FCRLA4d and FCRLA2d, respectively. The four-domain isoform was found to be much more abundant than the other isoforms, and SSP isoforms were about 10 times more abundant than LSP isoforms. Further investigation showed that LSP-FCRLA2d was a secreted isoform of the FCRLA protein [36], while all other isoforms were intracellular.

### Cellular expression of FCRL1 and association with immunoglobulins and disease

FCRL gene expression is seen primarily, although not exclusively, in B-lymphocytes. Expression of FCRL1 can be seen at low levels in pro- and pre-B cells and expression increases on naïve and memory B-cell populations. However, FCRL1 protein is predominantly expressed on the surface of mature B-cells, and is down regulated in germinal center (GC) B-cells [10,11,48]. A comparative analysis of FCRL1 gene expression showed that the expression level of FCRL1 in PBMCs is significantly higher in patients with autoimmune diseases like multiple sclerosis, lupus anticoagulans, Takayasu's arteritis and also in von Willebrand disease as compared with the healthy controls.

FCRL2 and FCRL3 expression peaks on peripheral memory B-cells and they also designate a population of circulating innate-like marginal zone (MZ) cells [14,16,49]. FCRL3 expression can also be seen in NK and CD8^+^ T-cells as well as Treg cells [11]. FCRL4-expressing B-cells show characteristics similar to MZ B-cells, and can be found in the mucosa-associated lymphoid tissue (MALT) lymphoma populations. These cells are a sub-population of memory B-cells that are tissue-based and also have an activated phenotype [17,19,50]. FCRL5 is widely expressed among B-cells and is highly expressed on plasma B-cells in the bone marrow, tonsils, and spleen. It is also expressed at low levels on pre-B cells and its expression increases on naïve and memory B cells [11]. Like FCRL3, FCRL6 expression is found on cytolytic lymphocytes, primarily on the surface of NK cells and also on CD8^+^ T cells in the blood and spleen [9,20,21].

Unlike FCRL3, FCRL6 is not typically expressed on human B-cells. A recent study suggested that FCRL6 are expressed in antigen-sensitized T cells [9]. On the other hand, Wilson et al. showed that FCRL6 expression is down-regulated in anti-CD3 or cytokine activated T cells [20]. This study also analyzed if mitogen stimulation alters FCRL6 gene expression in B and T cells [9], and found that mitogenic stimulation had no significant effect on FCRL6 gene expression in these cells, suggesting that antigen-specific activation may influence FCRL6 expression in T and B cells modulating immune responses in the host. Interestingly, FCRL6 gene expression is also upregulated in lymphocytes from HIV-1 infected individuals. Kulemzin et al. also found that FCRL6 gene is predominantly detected in T cells rather than B cells when analyzed patient samples from autoimmune diseases like rheumatoid arthritis (RA), systemic lupus erythematosus (SLE), and idiopathic thrombocytopenia purpura (ITP). Since FCRL6 is shown to be a ligand for human HLA-DR [26], the expression of FCRL6 in various cell types may regulate immunity in the host.

Expression of FCRL1-FCRL5 in B-cells increases during B-cell differentiation and peaks in circulating cells, as well as, cells localized to secondary lymphoid tissues, according to Northern blot, PCR, and in situ hybridization studies [4,5]. As for the intracellular FCRL proteins, FCRLA expression is found on most B-cell subsets in the tonsil and peripheral blood, and is highest in GC B-cells [22], most noticeably in the proliferating centroblasts, but is mainly absent in terminally differentiated plasma B-cells [51]. Because FCRLB expression is typically low, it is somewhat difficult to detect using Northern blot and RT-PCR. Therefore, expression could only be seen after amplification where it was found in the placenta, kidney and spleen [44]. Of the eight human FCRL proteins, six of them are surface proteins with a transmembrane region, while two of them are exclusively intracellular proteins. The surface FCRLs (FCRL1-FCRL6) express extracellular Ig-like domains, and ITIM- or ITAM-like sequences in their cytoplasmic tails, which suggests their involvement in immune regulation of their respective cell types. FCRLA and FCRLB, on the other hand, are expressed mainly intracellularly, and do not contain the ITIM- or ITAM-like sequences. They do, however, have C-termini containing a proline-rich stalk region which precedes a leucine-rich coiled-coil motif [24].

A recent study examined the binding ability of human FCRL proteins to immunoglobulins and found that FCRL4 bound to IgA whereas FCRL5 differentially bound to all IgG isotypes (Wilson 2013, JI), suggesting that these molecules may regulate B-cell function and could serve as therapeutic targets for various immune-mediated diseases. While FCRL3 weakly binds to IgG3, FCRL6 is considered as a ligand for HLA-DR. Association of FCRLA with IgG, IgM, and IgA has been shown to be occurred intracellularly suggesting its role as a possible chaperone protein in modulating cellular functions [24]. Furthermore, studies showed that cell surface FCRLA failed to bind extracellular Ig (Wilson 2010, Santiago 2011), suggesting this protein may be unstable when expressed on the cell surface, and could be involved in the development of B-cell lymphomas.

### FCRLs in cellular signaling

There are no known ligands for FCRL1-FCRL3 or FCRLB; however, ligands for the other family members have been discovered. Due to their homology to FcRs, FCRL4 and FCRL5 were tested to see if they could bind IgA and IgG. FCRL5 was shown to interact with IgG when stained with a preparation of mixed isotypes [11]. This was confirmed by using flow cytometry-based Ig-binding studies using FCRL1–FCRL6 transient transfectants. It was found that FCRL4 could bind to heat-aggregated IgA, while FCRL5 could bind to both heat-aggregated IgA and IgG [18]. This makes FCRL4 the only known receptor to inhibit IgA function. Wilson et al. also found that FCRL5 had greater affinity towards IgG1 and IgG2 than IgG3 and IgG4 [18]. Additionally, of its nine domains, the three N-terminal domains were found to be sufficient for reactivity. This was tested using cDNA encoding different domains, which were then expressed in cells and analyzed for the ability to bind Ig. Further investigation showed that using receptor specific mAbs could block binding of FCRL4 and FCRL5 with Igs. This verified these domains as being necessary for antibody binding and reinforces the roles of FCRL4 and FCRL5 as Ig receptors [18].

Schreeder et al. also found that FCRL6 can bind to MHC molecules, specifically HLA class II protein [21]. Due to its expression on NK cells and cytotoxic T cells, this study was performed using an engineered NFAT driven GFP containing cell line co-transduced with the human FCRL6 extracellular region fused to the CD3ζ cytoplasmic tail of an ITAM-bearing mouse [26]. Based on variable staining of FCRL6 in MHC class II expressing transductants, these studies also revealed that the binding affinities could differ between haplotypes of MHC II. While NK cells and cytotoxic T cells play vital roles in maintaining cell-mediated immunity, MHC class II-expressing B-cells, macrophages and dendritic cells can influence helper T cell responses, and the interaction of FCRL6 with MHC class II could prove to be a critical pathway involved in regulating innate as well as adaptive immune responses in the host.

Similarly, there have also been ligands discovered for intracellular FCRLA. Despite the fact that early studies failed to show interactions with immunoglobulins, two of its domains bear a resemblance to two of the three subunits in the CD64/FcγR1. Furthermore, immunoprecipitation studies with endogenous FCRLA showed that it co-associates with IgM, IgG, and IgA in studies with B-cell lines and primary B-cells [24]. These findings, combined with its expression patterns in germinal center B-cells could suggest its involvement in Ig retention during affinity maturation [52]. Although, FCRLB is even more similar to CD64, there have been no similar results suggesting Ig binding [24].

### FCRLs in immune regulation

#### FCRLs in innate immunity

A number of the FCRL proteins have been shown to have a significant role in the innate immune system. Beginning with FCRL3, there has been work done which has shown its ability to promote B-cell activation induced by toll-like receptor 9 (TLR9) which suppresses plasma cell differentiation [16]. It has also been indicated that FCRL3 is a trigger for B-cell proliferation, survival, as well as the induction of CD25, CD86, and HLA-DR activation markers. FCRL3 has also been shown to inhibit the production of immunoglobulin. Furthermore, differentiation of antibody secreting cells was interrupted and B-cell proliferation was promoted when transitional B-cells were cultured with CpG and FCRL3. Further investigation via flow cytometry revealed that FCRL3 did, in fact, improve stimulation of CpG-mediated NF-κB p65 and MAPK pERK and p38 [52], suggesting its implication in modulating innate immunity.

FCRL4 is also involved in modulating innate immunity as it binds to pTyr, SHP-1, and SHP-2 [53], impairs immune synapse formation, and blocks antigen-induced BCR signaling. It can halt induction of CD69 activation marker while upregulating activation associated protein CD23 with co-ligation of the BCR. Furthermore, it was noted that all of the ITAM and ITIM residues were necessary for FCRL4-mediated inhibition of Syk activation as well as the activation of PLCγ2, Vav, and the calcium signaling pathways. FCRL5 plays a role in innate immunity by its ability to be induced by anti-Ig co-stimulation. Additionally, mAb-directed co-ligation of FCRL5 and the BCR shared with anti-CD40 and IL-2 stimulation enhanced the proliferation of B-cells during TLR9 stimulation [54]. In hairy cell leukemia, there is also a phenomenon in which FCRL5 expression caused IgG and IgA isotype switching in addition to a group of abnormal cells that co-expressed multiple Ig isotypes [55]. However, these findings do conflict with other findings that show FCRL3’s inhibitory function on the secretion of IgG as well as generation of plasma cells [52].

#### FCRLs in adaptive immunity

In addition to innate immunity, the FCRL proteins play a significant role in adaptive immunity. As previously mentioned, most of the FCRL family members contain both ITAM and ITIM motifs in their cytoplasmic tails. This suggests that they could engage in dual modulation of B-cells. For example, FCRL3 has the ability to recruit Syk, Zap-70, SHP-1 and SHP-2 [56], and modulate cellular immune responses. Also, crosslinking of the BCR with FCRL2–5 stimulates their pTyr and docking phosphatases (SHP1 and/or SHP-2 SH2) at the consensus ITIMS sequences. Thus, receptor-mediated MAPK activation and calcium mobilization is weakened. In addition, studies were done in which non-functional FCRL2–FCRL5 ITIMs were transfected into a B-cell line and this caused an increase in calcium influx compared with the BCR engagement on its own [52], suggesting that FCRLs may regulate both humoral and cell mediated immune responses.

FCRL1 is an exception from the other FCRL proteins in that it contains two ITAM-like sequences, suggesting its role as a co-activation molecule. Thus far, it has been established that increased calcium flux and B-cell proliferation signifies activation via crosslinking FCRL1 with the BCR. Also, B-cell proliferation and FCRL1’s pTyr can be triggered via ligation mediated by receptorspecific mAbs [10]. Interestingly, FCRL1 also differs from the other family members in that it also contains a charged glutamic acid residue in its transmembrane region. Frequent expression of FCRL1 as well as other FCRLs has been detected in non-Hodgkin’s B-cell lymphoma (NHL) cell lines and patient samples [13,21,40], indicating that FCRL may represent a potential marker of B-cell tumors. While the role of FCRLs in the pathogenesis of autoimmune diseases remains unclear, a recent study reported downregulation of FCRL1 and upregulation of FCRL2 transcripts in Hashimoto's thyroiditis (HT) and Graves' disease (GD) [57]. In that study, overexpression of FCRL4 was observed in GD patients as compared to healthy controls. A significant correlation was also observed in all FCRLs gene expression in HT patients, while FCRLs 2 and 4 had a correlation in GD patients. A recent study also showed a significant decrease in the FCRL6 gene expression in peripheral T cells of patients with certain autoimmune and blood diseases [9], and its upregulation in patients with late stage HIV infection, suggesting FCRL6’s inhibitory potential in the modulation of immune effector functions in infectious diseases.

### FCRLs in pathophysiology of B-cell-associated diseases

Since the discovery of the FCRL proteins, their involvement in a number of different immune mediated malignancies has been reported for the different family members. As expected, the expression of these proteins is particularly prevalent in B-cell malignancies. Upon searching for FCRL sequences using the Lymphochip microarray database, FCRLs were found to be upregulated in DLBCL, FL, and CLL [58]. These results were later confirmed using mAbs for FCRL1–FCRL5, where protein expression was found on the surface of the previously mentioned lymphomas as well as BL, HCL, and MCL [11]. Because of their differential expression on different malignancies, FCRL proteins are also being investigated as diagnostic markers as well as targets for immunotherapies. These proteins, therefore, play critical roles in the understanding and treatment of malignant diseases.

FCRL1 is expressed widely among B-cells and therefore could show promise as an immunotherapeutic target. Further investigation revealed that FCRL1 expression was found largely on CLL, FL, HCL, and MCL samples. These findings led to cytotoxicity studies where two recombinant immunotoxins E3(Fv)-PE38 and E9(Fv)-PE38 were constructed, and found to be cytotoxic to lymphoma cell lines [13]. The family member, FCRL2, has emerged as an important molecule in CLL. Somatic hypermutation in the heavy chain variable region (IGHV) gene differentiates the subtypes of the disease based on aggressiveness. FCRL2 also became an important factor when it was found to be 94% concordant with IGHV status [59]. This made it a better biomarker than those that have been previously used.

The FCRL3 protein has been found to be prevalent in autoimmune disorders [16]. When the region surrounding the FCRL1-FCRL5 locus was surveyed, there was a number of single nucleotide polymorphisms (SNP) discovered. One of these SNPs was associated with 830 individuals with rheumatoid arthritis, systemic lupus erythematosus, Grave’s disease, as well as other autoimmune disorders [29]. Further investigation identified the principle variant was located in the FCRL3 promoter region in a possible NFκB binding motif. In more recent studies, there has been indication that FCRL3 modulation is associated with this SNP in T-cells and is also related to the clinical progression of rheumatoid arthritis [60]. Because of FCRL3’s association with these autoimmune diseases, the gene has now been considered as a candidate susceptibility gene for autoimmunity [52]. A recent study has also shown that FCRL4-expressing B-cells express high levels of TNF-α and RANKL, and possess pro-inflammatory roles in Rheumatoid arthritis [61].

Because FCRL4 expression marks a specific subset of B-cells that exist near the epithelium in MALT, it was investigated as a possible marker for MZL. Consequently, the protein was observed in 73% of nodal, and 93% in extranodal MZL making it an ideal histopathological marker for these malignancies [30]. However, it was not found to be in the specific subtype which stems from the spleen. FCRL4 also has a role in infectious diseases, where its expression has emerged in associated with diseases such as HIV and Hepatitis C [27,28]. In addition, a population of FCRL4-expressing B-cells was found in patients with combined variable immunodeficiency. A unique subset of B-cells in viremic HIV patients was also found to co-express FCRL4. In addition, it has been shown that FCRL4 knockdown via siRNA could re-establish BCR-mediated proliferation, production of cytokines and chemokines, and antibody responses specific to HIV [62]. However, it remains unclear how FCRL4 is involved with chronic viral infections.

FCRL5 has been shown to have a significant role in B-cell malignancies as well [52]. Patients with various B-cell malignancies were found to have soluble FCRL5 in their sera [63]. Importantly, FCRL5 is being studied as a possible target for MM immunotherapy. The expression of FCRL5 on MM plasma calls was established via analysis of bone marrow aspirates from MM patients. Thus far, antibody-drug conjugates have been developed targeting FCRL5, and they have shown potential in preclinical efficacy for targeting MM in xenograft models [31]. FCRL6 has also been implicated in immune disorders as gene expression has been shown to be upregulated in the late stages of HIV-1 infection [9]. In a recent study, RT-PCR was utilized to analyze the expression levels of FCRL6 in peripheral blood lymphocytes of late-stage HIV-1 infected patients, where expression levels were significantly higher in stage 3 and 4 HIV-1 infected patients when compared with healthy individuals [9]. This study suggests that FCRL6 may influence HIV infection, and further studies may find ways to be able to utilize it in diagnosis or treatment of HIV infection.

## Conclusions

Until recently, there was little information available about the FCRL protein family, but the progress being made is beginning to show their critical roles in the human immune system. Beginning with their cellular expression and structural components which highly suggest modulation of FCRLs primarily in B-cell populations, but also in other subsets of immune cells, these proteins show strong potential for immune regulation. Recent findings which have uncovered ligands for a number of the FCRL proteins will also allow investigators for a better understanding of their signaling events and functions. Thus far, the FCRL proteins have shown roles in both adaptive and innate immune functions with much more that is still unknown about their functionality. Their presence and roles in the immune system reveal themselves in their association with lymphoproliferative diseases, autoimmunity, cancer, as well as chronic viral diseases. This makes these proteins ideal as markers and/or targets in diagnosis, prognosis, as well as treatment for these diseases. Further investigation of these proteins could lead to major breakthroughs in a number of human diseases including lymphomas, viral infections, and autoimmunity.

## Figures and Tables

**Table 1:** Summary of Fc receptor-like proteins. This chart gives a summary of each FCRL protein and its known ligands, cellular distribution, and association with different diseases.

	Ligands	Cellular Distribution	Associated Diseases
FCRL1	Unknown	Broad B cell expression [10,11]	Burkitt Lymphoma CLL
FCRL2	Unknown	Memory B cells [11] Circulating Marginal Zone B cells	IGHV CLL
FCRL3	Unknown	Memory B cells [16] Circulating Marginal Zone B cells [16] T cells [11] NK cells C	Autoimmunity
FCRL4	Heat-aggregated IgA	Memory B cells Tissue-based Marginal Zone B cells [17]	HIV Hepatitis C MZ Lymphomas
FCRL5	Heat-aggregated IgG	Naive B Cells [11]Memory B Cells [11] Plasma Cells [11]	Burkitt Lymphoma
FCRL6	MHC Class II/HLA-DR, Intracellular IgM and IgG	Cytotoxic T cells [22] NK Cells [22]	HIV-1
FCRLA	Intracellular IgM, IgG, and IgA	Germinal Center B cells	Unknown
FCRLB	Unknown	Germinal Center B cells	Unknown

**Table 2:** Summary of nomenclature for Fc receptor family members. This chart summarizes the new nomenclature for the FCRL proteins and their accession numbers in association with their previously identified names.

New Nomenclature	Previous Nomenclature	Accession Number
FCRL1	FcRH1 [5], IRTA5 [3], IFGP1 [6], BXMAS1 [7]	Q96LA6
FCRL2	FcRH2 [5], IRTA4 [3], IFGP4 [6], BXMAS2 [7], SPAP1 [41]	Q96LA5
FCRL3	FcRH3 [5], IRTA3 [3], IFGP3 [6], BXMAS3 [7], SPAP2 [41]	Q96P31
FCRL4	FcRH4 [5], IRTA1 [3], IFGP2 [6]	Q96PJ5
FCRL5	FcRH5 [5], IRTA2 [3] IFGP5 [6], BXMAS [7]	Q96RD9
FCRL6	FcRH6 [5], IFGP6 [7]	Q6DN72
FCRLA	FCRL [42], FREB [43], FcRX [3]	Q7L513
FCRLB	FcRL2 [42], FREB2 [44] FcRY [46]	Q6BAA4
